# Supplementary material for: Inter-regional interactions uncouple within-region inhibition stabilization and paradoxical responses
Source: bioRxiv. 2026 Sep 15:2026.09.09.750483. Preprint. [Version 1] doi: 10.64898/2026.09.09.750483 (PMC13596579; doi:10.64898/2026.09.09.750483)
Supplement: Supplementary file 1 [file NIHPP2026.09.09.750483V1-supplement-1.pdf]

bioRxiv preprint doi: https://doi.org/10.64898/2026.09.09.750483; this version posted September 15, 2026. The copyright holder for this preprint (which was not certified by peer review) is the author/funder, who has granted bioRxiv a license to display the preprint in perpetuity. It is made available under a CC-BY 4.0 International license.

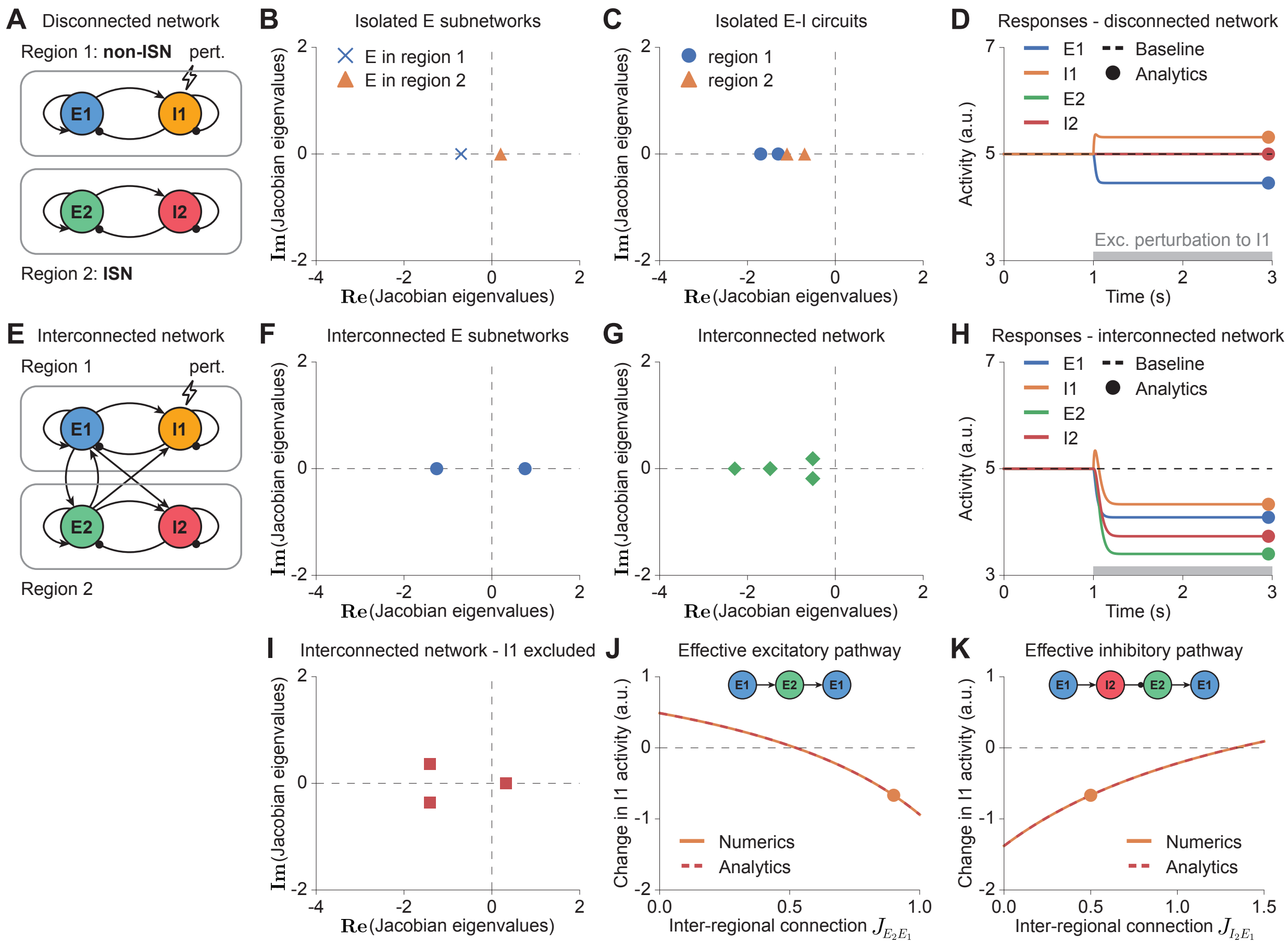

Fig. S1. Inter-regional connections can induce a paradoxical inhibitory response in a non-ISN coupled to an ISN. Panel organization, perturbation protocol, and plotting conventions follow Figure 1. **A–D.** Disconnected configuration. In isolation, region 1 is a non-ISN, whereas region 2 is an ISN. I_1 activity increases nonparadoxically in response to the excitatory perturbation. **E–K.** Interconnected configuration. **E–H.** Inter-regional coupling reverses the I_1 response to a paradoxical decrease while resulting in one unstable mode in the coupled excitatory subnetwork and preserving overall network stability. **I.** The interconnected network with I_1 excluded has an odd number of unstable eigenmodes (one in this case), consistent with the paradoxical response in **H**. **J–K.** The unperturbed region (region 2) has zero (an even number of) unstable eigenmodes, as shown in **C**. Increasing J_{E2E1}, which contributes to positive inter-regional feedback, decreases the perturbation-induced change in I_1 activity, whereas increasing J_{I2E1}, which contributes to negative inter-regional feedback, increases this change.

bioRxiv preprint doi: https://doi.org/10.64898/2026.09.09.750483; this version posted September 15, 2026. The copyright holder for this preprint (which was not certified by peer review) is the author/funder, who has granted bioRxiv a license to display the preprint in perpetuity. It is made available under a CC-BY 4.0 International license.

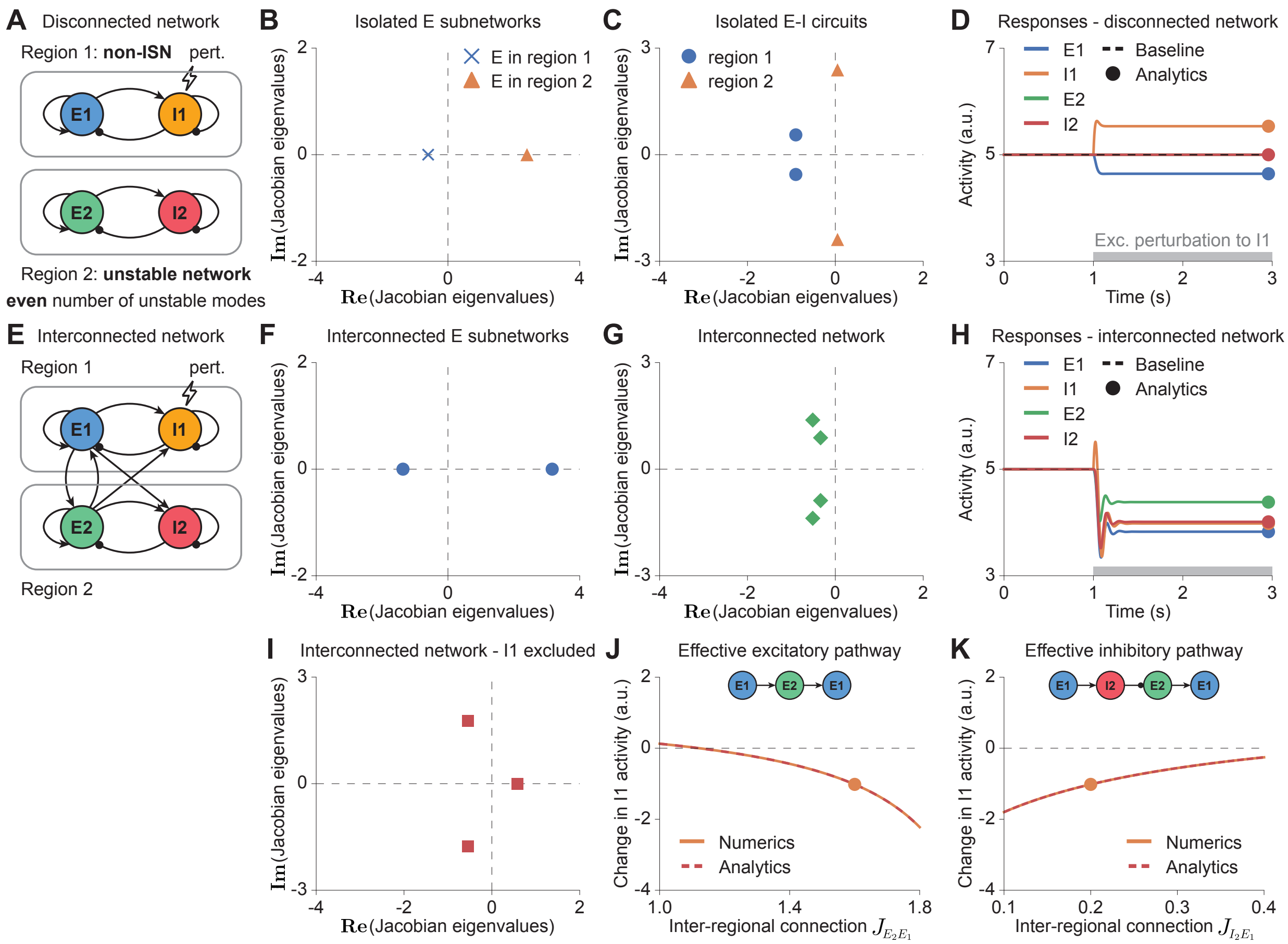

Fig. S2. Inter-regional connections can induce a paradoxical inhibitory response in a non-ISN coupled to an unstable network with an even number of unstable eigenmodes. Panel organization, perturbation protocol, and plotting conventions follow Figure 1. **A–D.** Disconnected configuration. In isolation, region 1 is a non-ISN, whereas region 2 is an unstable network with two (an even number of) unstable eigenmodes. I_1 activity increases nonparadoxically in response to the excitatory perturbation. **E–K.** Interconnected configuration. **E–H.** Inter-regional coupling reverses the I_1 response to a paradoxical decrease while resulting in one unstable mode in the coupled excitatory subnetwork and stabilizing the full interconnected network. **I.** The interconnected network with I_1 excluded has an odd number of unstable eigenmodes (one in this case), consistent with the paradoxical response in **H**. **J–K.** The unperturbed region (region 2) has two (an even number of) unstable eigenmodes, as shown in **C**. Increasing J_{E2E1}, which contributes to positive inter-regional feedback, decreases the perturbation-induced change in I_1 activity, whereas increasing J_{I2E1}, which contributes to negative inter-regional feedback, increases this change.

bioRxiv preprint doi: https://doi.org/10.64898/2026.09.09.750483; this version posted September 15, 2026. The copyright holder for this preprint (which was not certified by peer review) is the author/funder, who has granted bioRxiv a license to display the preprint in perpetuity. It is made available under a CC-BY 4.0 International license.

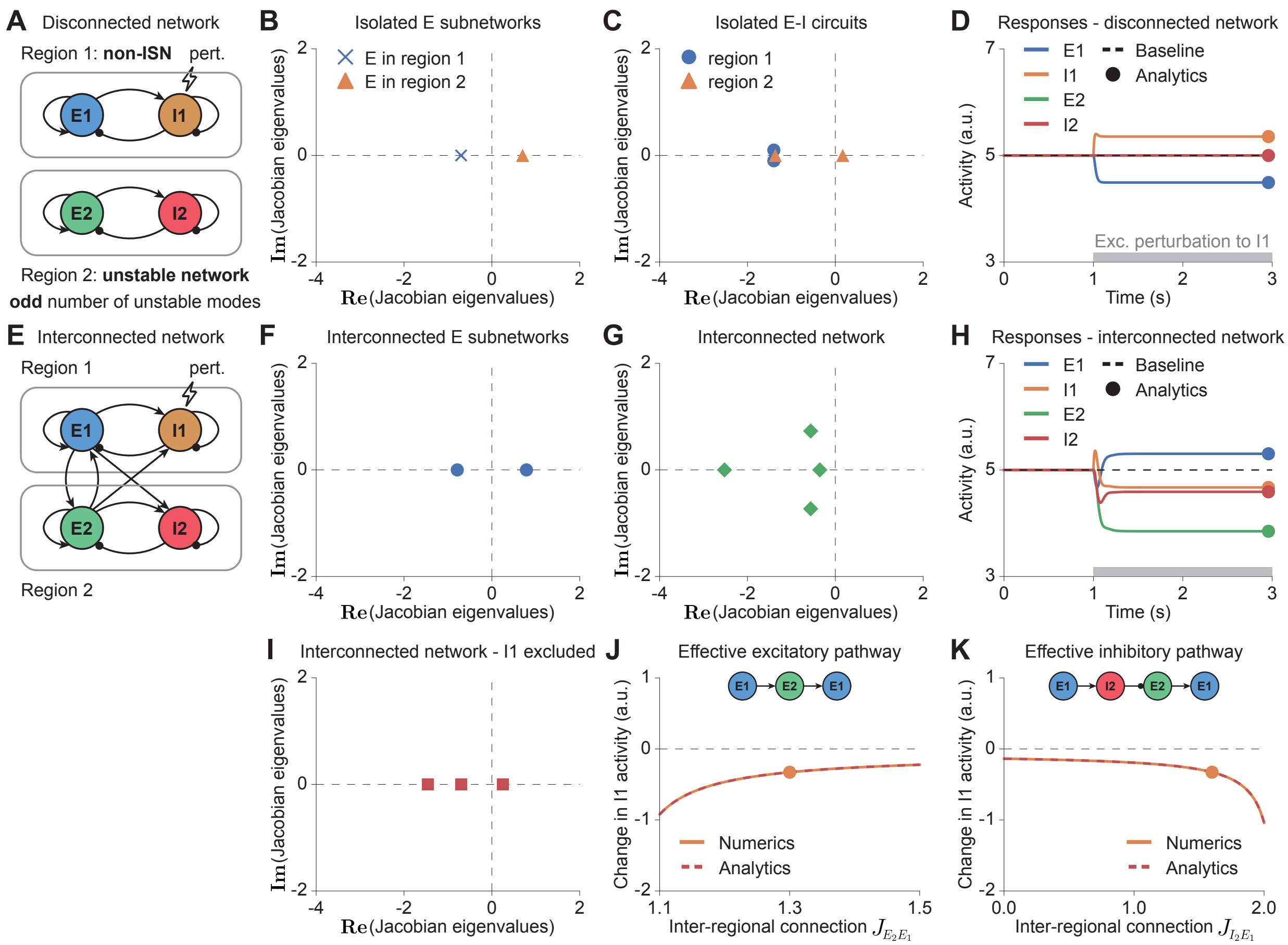

Fig. S3. Inter-regional connections can induce a paradoxical inhibitory response in a non-ISN coupled to an unstable network with an odd number of unstable eigenmodes. Panel organization, perturbation protocol, and plotting conventions follow Figure 1. **A–D.** Disconnected configuration. In isolation, region 1 is a non-ISN, whereas region 2 is an unstable network with a single unstable eigenmode and thus an odd number of unstable eigenmodes. I_1 activity increases nonparadoxically in response to the excitatory perturbation. **E–K.** Interconnected configuration. **E–H.** Inter-regional coupling reverses the I_1 response to a paradoxical decrease while resulting in one unstable mode in the coupled excitatory subnetwork and stabilizing the full interconnected network. **I.** The interconnected network with I_1 excluded has an odd number of unstable eigenmodes (one in this case), consistent with the paradoxical response in **H**. **J–K.** The unperturbed region (region 2) has an odd number of unstable eigenmodes (one in this case), as shown in **C**. Unlike in Figures 1, S1, and S2, increasing J_{E2E1}, which is involved in the effective excitatory pathway $E_1 \rightarrow E_2 \rightarrow E_1$, increases the perturbation-induced change in I_1 activity, whereas increasing J_{I2E1}, which is involved in the effective inhibitory pathway $E_1 \rightarrow I_2 \rightarrow E_2 \rightarrow E_1$, decreases this change.

bioRxiv preprint doi: https://doi.org/10.64898/2026.09.09.750483; this version posted September 15, 2026. The copyright holder for this preprint (which was not certified by peer review) is the author/funder, who has granted bioRxiv a license to display the preprint in perpetuity. It is made available under a CC-BY 4.0 International license.

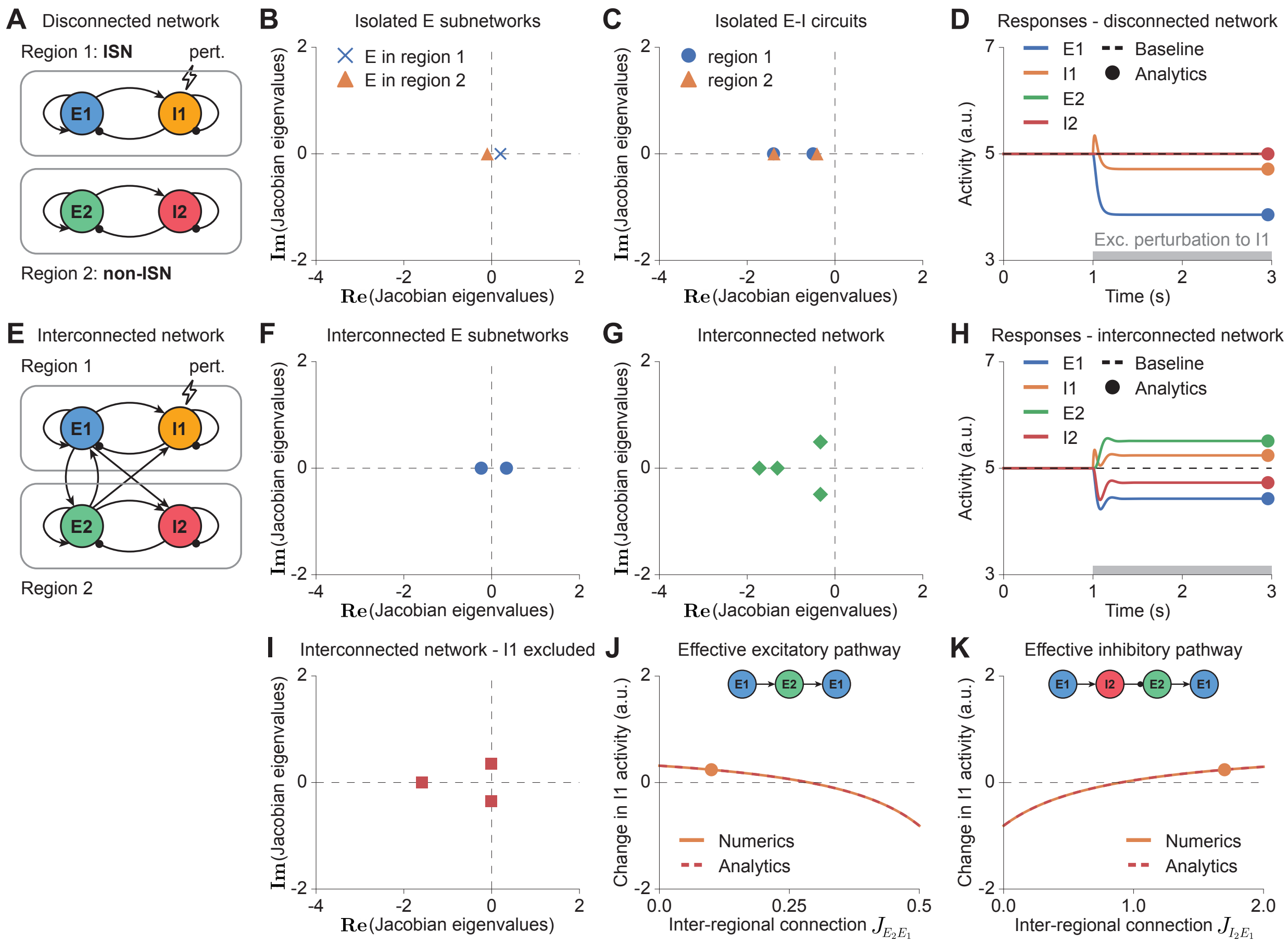

Fig. S4. Inter-regional connections can abolish the paradoxical inhibitory response in an ISN coupled to a non-ISN. Panel organization, perturbation protocol, and plotting conventions follow Figure 1. **A–D.** Disconnected configuration. In isolation, region 1 is an ISN, whereas region 2 is a non-ISN. I_1 activity decreases paradoxically in response to the excitatory perturbation. **E–K.** Interconnected configuration. **E–H.** Inter-regional coupling reverses the I_1 response to a nonparadoxical increase while resulting in one unstable mode in the coupled excitatory subnetwork and preserving overall network stability. **I.** The interconnected network with I_1 excluded has zero (an even number of) unstable eigenmodes, consistent with the nonparadoxical response in **H**. **J–K.** The unperturbed region (region 2) has zero (an even number of) unstable eigenmodes, as shown in **C**. For the parameters shown, increasing J_{E2E1}, which contributes to positive inter-regional feedback, decreases the perturbation-induced change in I_1 activity, whereas increasing J_{I2E1}, which contributes to negative inter-regional feedback, increases this change.

bioRxiv preprint doi: https://doi.org/10.64898/2026.09.09.750483; this version posted September 15, 2026. The copyright holder for this preprint (which was not certified by peer review) is the author/funder, who has granted bioRxiv a license to display the preprint in perpetuity. It is made available under a CC-BY 4.0 International license.

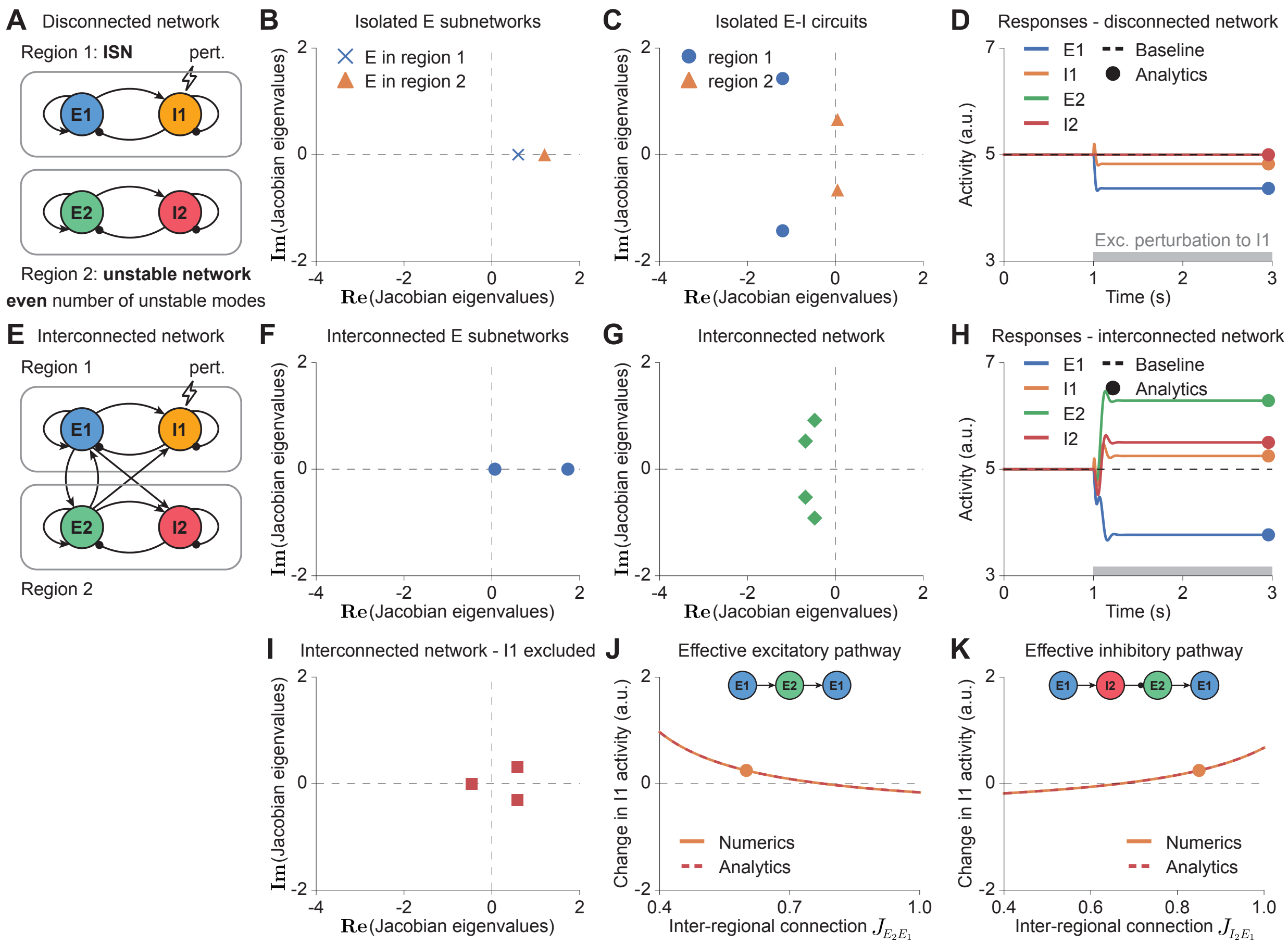

Fig. S5. Inter-regional connections can abolish the paradoxical inhibitory response in an ISN coupled to an unstable network with an even number of unstable eigenmodes. Panel organization, perturbation protocol, and plotting conventions follow Figure 1. **A–D.** Disconnected configuration. In isolation, region 1 is an ISN, whereas region 2 is an unstable network with two (an even number of) unstable eigenmodes. I_1 activity decreases paradoxically in response to the excitatory perturbation. **E–K.** Interconnected configuration. **E–H.** Inter-regional coupling reverses the I_1 response to a nonparadoxical increase while resulting in two unstable modes in the coupled excitatory subnetwork and stabilizing the full interconnected network. **I.** The interconnected network with I_1 excluded has two (an even number of) unstable eigenmodes, consistent with the nonparadoxical response in **H**. **J–K.** The unperturbed region (region 2) has two (an even number of) unstable eigenmodes, as shown in **C**. For the parameters shown, increasing J_{E2E1}, which contributes to positive inter-regional feedback, decreases the perturbation-induced change in I_1 activity, whereas increasing J_{I2E1}, which contributes to negative inter-regional feedback, increases this change.

bioRxiv preprint doi: https://doi.org/10.64898/2026.09.09.750483; this version posted September 15, 2026. The copyright holder for this preprint (which was not certified by peer review) is the author/funder, who has granted bioRxiv a license to display the preprint in perpetuity. It is made available under a CC-BY 4.0 International license.

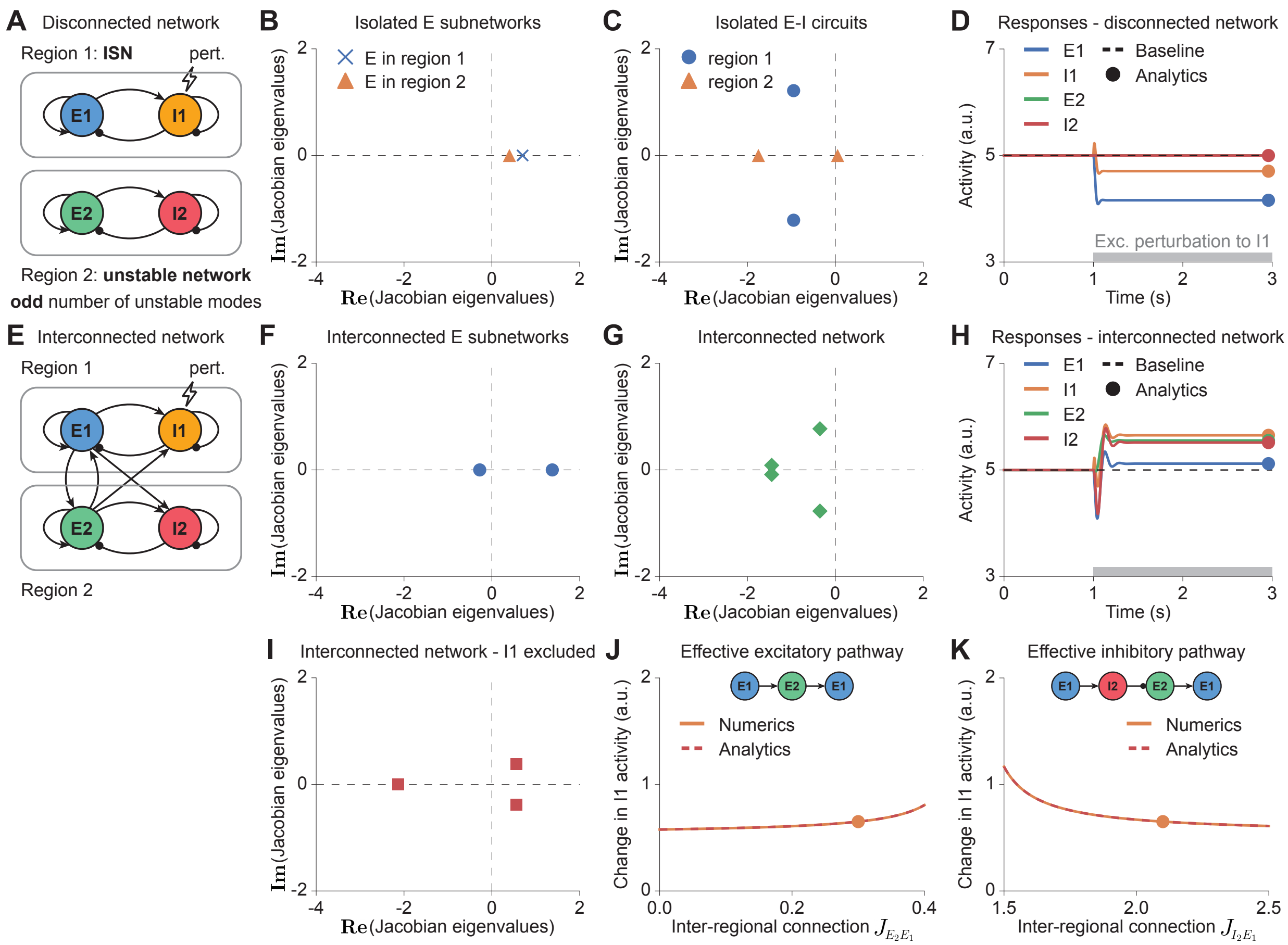

Fig. S6. Inter-regional connections can abolish the paradoxical inhibitory response in an ISN coupled to an unstable network with an odd number of unstable eigenmodes. Panel organization, perturbation protocol, and plotting conventions follow Figure 1. **A–D.** Disconnected configuration. In isolation, region 1 is an ISN, whereas region 2 is an unstable network with a single unstable eigenmode and thus an odd number of unstable eigenmodes. I_1 activity decreases paradoxically in response to the excitatory perturbation. **E–K.** Interconnected configuration. **E–H.** Inter-regional coupling reverses the I_1 response to a nonparadoxical increase while resulting in one unstable mode in the coupled excitatory subnetwork and stabilizing the full interconnected network. **I.** The interconnected network with I_1 excluded has two (an even number of) unstable eigenmodes, consistent with the nonparadoxical response in **H**. **J–K.** The unperturbed region (region 2) has an odd number of unstable eigenmodes (one in this case), as shown in **C**. For the parameters shown, unlike in Figures 2, S4, and S5, increasing J_{E2E1}, which is involved in the effective excitatory pathway $E_1 \to E_2 \to E_1$, increases the perturbation-induced change in I_1 activity, whereas increasing J_{I2E1}, which is involved in the effective inhibitory pathway $E_1 \to I_2 \to E_2 \to E_1$, decreases this change.
